# The role and therapeutic potential of macrophages in the pathogenesis of diabetic cardiomyopathy

**DOI:** 10.3389/fimmu.2024.1393392

**Published:** 2024-05-07

**Authors:** Shan Zhang, Xueying Zhu, Yupeng Chen, Zhige Wen, Peiyu Shi, Qing Ni

**Affiliations:** ^1^ Department of Endocrinology, Guang’anmen Hospital, China Academy of Chinese Medical Sciences, Beijing, China; ^2^ Department of Anatomy, School of Traditional Chinese Medicine, Beijing University of Chinese Medicine, Beijing, China

**Keywords:** macrophages, diabetic cardiomyopathy, diabetes, inflammation, cardiovascular diseases

## Abstract

This review provides a comprehensive analysis of the critical role played by macrophages and their underlying mechanisms in the progression of diabetic cardiomyopathy (DCM). It begins by discussing the origins and diverse subtypes of macrophages, elucidating their spatial distribution and modes of intercellular communication, thereby emphasizing their significance in the pathogenesis of DCM. The review then delves into the intricate relationship between macrophages and the onset of DCM, particularly focusing on the epigenetic regulatory mechanisms employed by macrophages in the context of DCM condition. Additionally, the review discusses various therapeutic strategies aimed at targeting macrophages to manage DCM. It specifically highlights the potential of natural food components in alleviating diabetic microvascular complications and examines the modulatory effects of existing hypoglycemic drugs on macrophage activity. These findings, summarized in this review, not only provide fresh insights into the role of macrophages in diabetic microvascular complications but also offer valuable guidance for future therapeutic research and interventions in this field.

## 1 Introduction

Diabetes Mellitus (DM) is a chronic metabolic disorder characterized by persistently elevated blood glucose levels (1). Reports from 2019 estimate the global prevalence of diabetes to be 9.3% (463 million individuals), with projections suggesting an increase to 10.2% (578 million individuals) by 2030 (2). Among the cardiovascular complications induced by diabetes, microvascular disease, primarily in the form of DCM, is often diagnosed in its later stages and is considered a leading cause of heart failure in diabetic patients (3, 4). Macrovascular complications primarily refer to accelerated atherosclerosis, including coronary artery disease (CAD) that can lead to acute myocardial infarction (MI) (5). A low-grade inflammatory state (metabolic inflammation) is one of the hallmarks of DCM and the accelerated atherosclerosis associated with diabetes (6, 7). In sites of chronic inflammation in diabetic patients, monocytes/macrophages are excessively recruited, playing an indispensable role in DCM (8).

This article delves into the mechanisms by which macrophages contribute to the development and progression of DCM, as well as the impact of existing therapeutic strategies on these cells. Through a comprehensive analysis of these key mechanisms and cell types, the article aims to offer new perspectives and strategies for the prevention and treatment of diabetic cardiovascular complications, thereby aiming to enhance the quality of life and prognosis for individuals with diabetes.

## 2 Cardiac macrophages: origin, heterogeneity, and spatial niches

### 2.1 Origin of macrophages

In 1968, Van Furth and Cohen first defined macrophages in mice as being derived entirely from blood monocytes (9). It was not until 1984 that they discovered macrophage populations in the spleen to be independent of monocyte influx, thus bringing tissue-resident macrophages into focus (10). The mammalian heart contains a significant number of cardiac tissue-resident macrophages (cMacs). The epigenetic mechanisms dependent on the environment are one of the bases for regulating the transcriptional differences among heterogeneous subgroups of cMacs (11). Previous studies have identified three distinct subgroups of human cardiac macrophages based on markers such as C-C chemokine receptor type 2 (CCR2) and HLA-DR (a human major histocompatibility complex II [MHC-II] molecule), namely CCR2^+^ HLA-DR^low^, CCR2^+^ HLA-DR^high^, and CCR2^−^ HLA-DR^high^ (12). Furthermore, based on markers like lymphocyte antigen 6 complex, locus C (Ly6C), and MHC-II, four distinct subgroups of mouse macrophages can be effectively distinguished (13).

**Figure 1:**
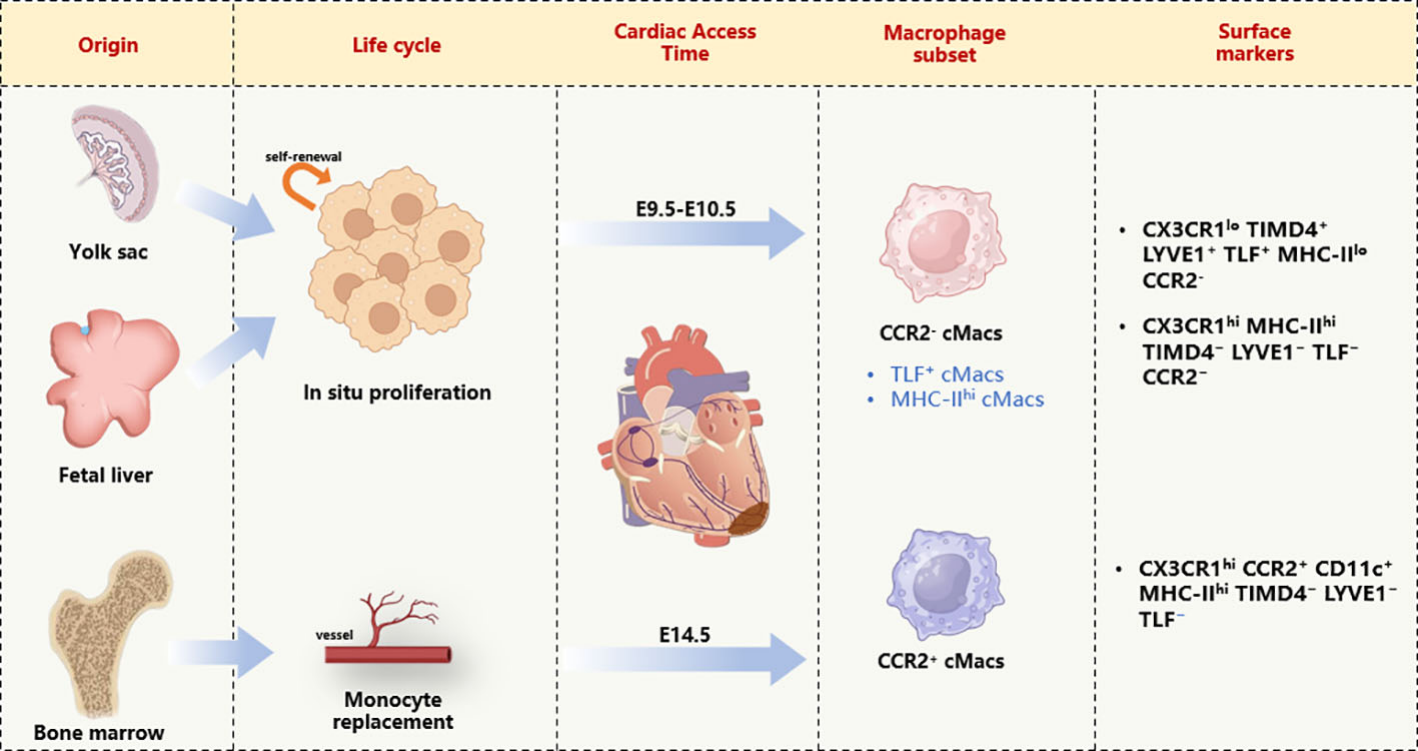
Cardiac macrophage diversity on the basis of developmental origin, life cycle, and transcriptional signature. cMacs, Cardiac resident macrophages; CD11c, Integrin alpha X; CCR2, CC- chemokine receptor 2; CX3CR, Chemokine (C-X3-C motif) receptor; LYVE1, lymphatic vessel endothelial hyaluronan receptor 1; MHC-II, major histocompatibility complex class II; TLF, folate receptor beta; TIMD4, T cell immunoglobulin and mucin domain containing 4.

With the advent of new technologies such as genetic fate mapping and lineage tracing, we are now able to label, track, and monitor the origins and developmental processes of macrophages in tissues (14). The earliest macrophage precursor cells originate from the extraembryonic yolk sac between embryonic day 7.0 (E7.0) and E9.0. As embryonic vasculature and the aorta-gonad-mesonephros (AGM) region develop, hematopoietic stem cells (HSCs) are produced and eventually colonize the fetal liver and bone marrow. Thus, at least two mechanisms (yolk sac and fetal liver) are responsible for establishing cMacs before birth (15). Monocyte-derived M1 macrophages primarily differentiate from blood monocytes produced by bone marrow HSCs, under the stimulation of colony-stimulating factor (CSF) and granulocyte-macrophage CSF, developing into monocytes, pre-monocytes, and mature monocytes. Subsequently, they proliferate into the body’s epithelial and submucosal tissues, which are essential for immune surveillance, self-stability, and resistance to infection (16). The earliest yolk sac-derived M2 macrophages are mostly attributed to cMacs, appearing in cardiac tissue between E9.5 and E10.5, followed by CCR2^+^ cMacs from fetal monocyte precursor cells colonizing the heart by E14.5 (17) (**Figure 1**).

### 2.2 Heterogeneity of macrophages

The M1/M2 binary classification of macrophages, as initially described by Mills CD and colleagues, is based on their responses to different stimuli *In vitro* (18). M1 macrophages are typically associated with pro-inflammatory and antimicrobial activities, while M2 macrophages are generally linked to anti-inflammatory actions, tissue repair, and tumor-promoting effects. Although this classification has its limitations and does not fully capture the complexity and diversity of macrophage functions *in vivo*, it remains a useful framework for researching and understanding macrophage roles under various physiological and pathological conditions. Recent studies, including the work of Xue J et al., have proposed a ‘spectrum model’ of macrophage activation, which offers a more nuanced view of macrophage function across a continuum of activation states (19). This model underscores the importance of considering the full range of macrophage activation in understanding their roles in health and disease.

**M1 macrophages:** Their surface markers include HLA-DR, CD80, CD86, and CD197, and they produce pro-inflammatory cytokines (20). Typically, Th1 (interferon [IFN]-γ, tumor necrosis factor-[TNF]-α) or bacterial lipopolysaccharide (LPS) can induce and activate M1 polarization, leading to the release of a large amount of pro-inflammatory cytokines, including TNF-α, interleukins (IL)-1, IL-6, IL-12, IL-18, IL-23, and chemokine ligands CXCL-9 and CXCL-10. In T2DM, the islets undergo inflammation, with a significant accumulation of M1-like macrophages, although it remains unclear what triggers this inflammatory cascade. Recent studies report that islet macrophages and β-cells are frontline responders to systemic metabolic changes. Furthermore, M1-like macrophages produce pro-inflammatory cytokines that promote β-cell dysfunction, creating a vicious cycle with β-cell secretion of chemokines and M1-like macrophage-secreted cytokines, accelerating islet inflammation (21).

**M2 macrophages:** Based on multiple activation signals received by M2 macrophages, four different M2 macrophage groups can be identified: M2a, M2b, M2c, and M2d. M2-like macrophages are maintained through the local proliferation of resident macrophages, playing an indispensable role in the proliferation of β-cells and participating in maintaining a healthy islet microenvironment (22). During the metabolic inflammation process in the islets, including macrophages, endocrine cells, and endothelial cells (ECs), jointly secrete a variety of cytokines, such as CSF-1, TGF-β1, and vascular endothelial growth factor-A (VEGFA), promoting the polarization of M2-type islet macrophages (23, 24). Tissue-resident macrophages belong to the M2 class, in addition, activated M2-type macrophages include anti-inflammatory macrophages, pro-fibrotic macrophages, and pro-angiogenic macrophages, which are involved in the proliferation of β-cells during development and in adult mice (25, 26) (**Table 1**).

**Table 1:** Phenotypes, stimulations, secretions, markers, and functional roles of M2 macrophages.

Phenotypes	Activation Signals	Surface Markers	Secreted Molecules	Functional Roles	Ref
M2a	IL-4, IL-13	CD206, CD209, CD301, CD163	IL-10, Mannose receptor CD206, Macrophage galactose type C lectin (MGL; CD301) Note: Arginase-1 is also involved in arginine metabolism and serves as a marker of M2a macrophages predominantly in mice, not in humans.	Eliminate pathogens, remove debris, stimulate angiogenesis, aid tissue regeneration, promote Th2 immunity	(27–30)
M2b	Immune complexes, TLRs, IL-1R ligands, LPS	CD206, CD209, CD301, CD163	High IL-10, Low IL-12, TNF-α, IL-1, IL-6	Reduce acute inflammation from bacterial endotoxins, promote Th2 differentiation and humoral immunity	(31)
M2c	CSF-1, IL-10, TGF-β, Glucocorticoids, Vitamin D, VEGFA	CD206, CD209, CD301, CD163	High TGF-β, High IL-10	Inhibit immunological inflammation	(32)
M2d/TAM	IL-6, Adenosine (in the context of tumors)	Lower F4/80, CD11b, CD68, CD115 than typical M2; More mannose receptor and scavenger receptor A than M1	IL-10, VEGF, Lower IL-1beta, IL-6, IL-12, TNF-alpha, ROI than M1	Inhibit immunity, promote tumor cell proliferation, stimulate angiogenesis, may have traits common to both M1 and M2 polarization, influenced by tumor microenvironment	(33, 34)

CD, Cluster of Differentiation; CSF-1, Colony Stimulating Factor-1; IFN-γ, Interferon-gamma; IL, Interleukin; LPS, Lipopolysaccharide; MGL, Macrophage Galactose type C Lectin; ROI, Reactive Oxygen Intermediates; TAM, Tumor-Associated Macrophages; TGF-β, Transforming Growth Factor-beta; Th2, T helper cell type 2; TLR, Toll-Like Receptors; TNF-α, Tumor Necrosis Factor-alpha; VEGF, Vascular Endothelial Growth Factor.

### 2.3 Spatial niches and intercellular communication of cardiac macrophages

The heart’s structural complexity and its physiological and pathological environments dictate the distinct characteristics displayed by cMacs in their respective niches. The heart, being a highly vascularized organ, sees a significant accumulation of CCR2^-^ cMacs around blood vessels (17). These cMacs regulate vascular homeostasis through their interactions with smooth muscle cells and collagen by expressing lymphatic vessel endothelial hyaluronan receptor 1 (LYVE-1) (35, 36). Communication between macrophages and ECs via extracellular vesicles (EVs) leads to changes in macrophage phenotypes, resulting in reduced microvascular repair and compromised myocardial perfusion (37). This process, in turn, promotes atherosclerosis, chronic inflammation, and fibrosis (38). Furthermore, interactions between macrophages and ECs through small extracellular vesicles (sEVs) also play a role in diabetic complications. High glucose levels in pro-inflammatory macrophages induce the production of sEVs carrying elevated levels of IL-1β, inducible nitric oxide synthase, human antigen R (HuR), miR-21-5p, miR-486-5p, and TGF-β mRNA (39–43). These sEVs can effectively transfer pathogenic miRNAs to the subendothelium, leading to vasoconstriction, dysfunction, and inflammation, which further promote atherosclerosis and thrombosis (44). Conversely, sEVs derived from ECs containing MALAT-1 promote the polarization of anti-inflammatory macrophages (45, 46).

In the context of cardiac electrophysiology, confocal fluorescence microscopy has shown that CCR2- cMacs are abundantly present at the atrioventricular node (47). These cMacs connect with conducting cardiomyocytes through gap junctions involving connexin 43 (Cx43) and regulate the electrical activity of cardiomyocytes through periodic depolarization (47). cMacs can also indirectly prevent cardiac conduction block and ventricular arrhythmias. In models of right heart pressure overload (pulmonary hypertension), macrophage-derived amphiregulin (Areg) has been shown to prevent lethal arrhythmias and sudden death (48). Areg plays a crucial role in controlling the phosphorylation and translocation of cardiomyocyte Cx43; its deletion leads to the disassembly of gap junctions (48). Research by Simon-Chica et al. revealed a mirroring effect in the expression of ion channel subunits between cardiomyocytes and cMacs, including Kir2.1, Kv1.5, Kv1.3, Cx43, and Nav1.5, thereby accelerating the repolarization of cardiomyocytes (49). This suggests that changes in macrophage phenotypes in DCM could be a potential cause of the cardiac electrical remodeling often observed in patients with type 1 and type 2 diabetes (50).

## 3 Pathogenesis of DCM

**Figure 2:**
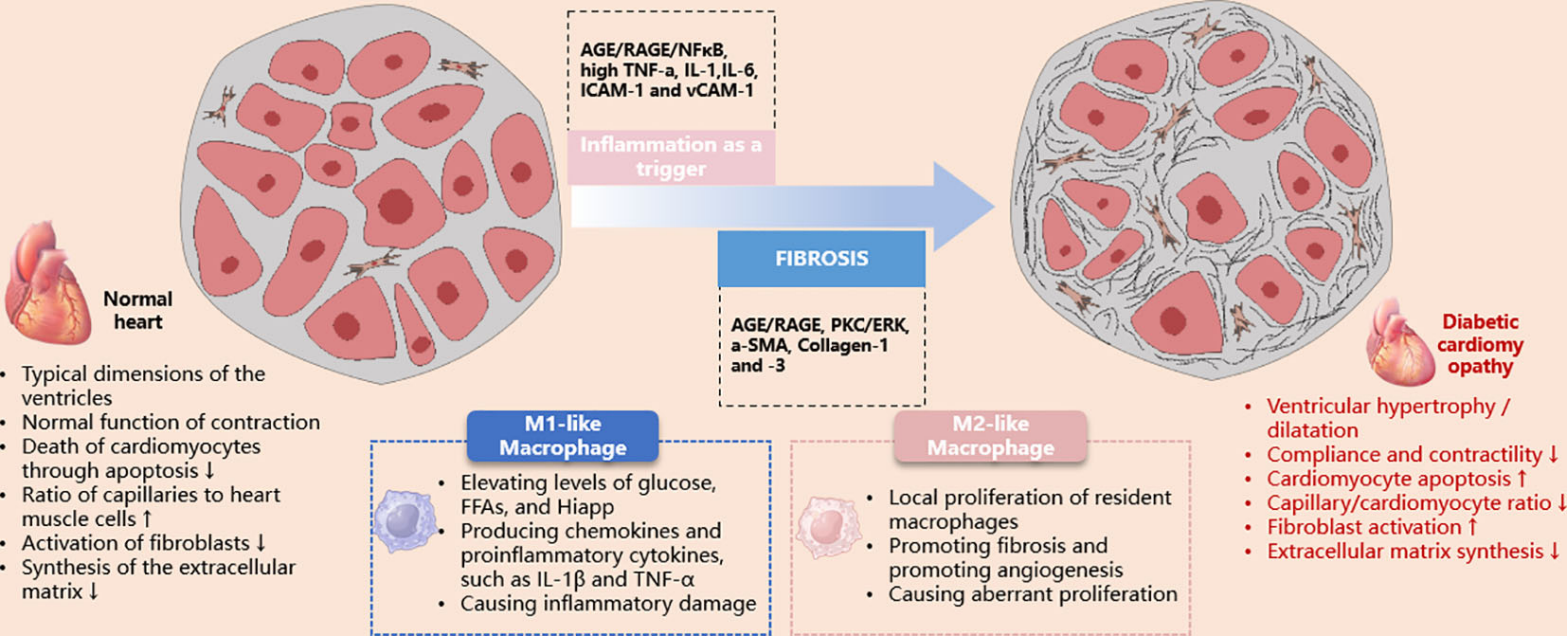
Schematic illustration of the role of macrophages in the pathological damage of diabetic cardiomyopathy. AGE, advanced glycation end products; ERK, extracellular signal-regulated kinase; ICAM-1, intercellular adhesion molecule-1; IL, interleukin; iNOS, inducible nitric oxide synthase; PKC, protein kinase C; RAGE, receptor for advanced glycation end products; TNF, tumor necrosis factor; VCAM-1, vascular cell adhesion molecule-1.

DCM is a myocardial-specific microvascular complication that occurs independently of CAD, valvular disease, and hypertension (51). It increases the risk of heart failure in diabetic patients (52). The overproduction of free radicals in a hyperglycemic environment has been identified as a key pathogenic factor in the development of DCM. This process induces oxidative stress, mitochondrial dysfunction, apoptosis, and myocardial fibrosis, thereby impairing myocardial contractility and normal cardiac function. Abnormal cellular metabolism and the accumulation of damaged or defective organelles are associated with the pathogenesis of DCM (53) (**Figure 2**).

### 3.1 Inflammation as a trigger for DCM

The involvement of the innate immune system and pro-inflammatory responses is associated with the development of DCM. The activation and expression of pro-inflammatory cytokines, such as TNFα, IL-6 and 8, monocyte chemoattractant protein 1 (MCP-1), intercellular adhesion molecule 1, and vascular cell adhesion molecule 1, contribute to cardiac oxidative stress, remodeling, fibrosis, and diastolic dysfunction (54). The expression of these cytokines is regulated by the nuclear transcription factor NF-κB (54). Additionally, Toll-like receptor 4 plays a significant role in triggering NF-κB activation, enhancing pro-inflammatory and innate immune responses (55). The increased migration of monocytes/macrophages into the coronary endothelium elevates resident cardiac macrophages. Under conditions of increased ROS and reduced bioavailable NO, these macrophages can polarize into a pro-inflammatory M1 phenotype (54–56). Recent studies have observed an upregulation of pro-inflammatory M1 polarization in diabetic cardiac tissue, while the M2 anti-inflammatory response is suppressed (57–61).

Beyond the apparent pro-inflammatory state in diabetes, the body’s attempt to recover from this persistent damage may also be impaired. Infiltrating macrophages exhibit defective efferocytosis of apoptotic neutrophils at sites of vascular inflammatory damage (62). Thus, defective resolution of inflammation may represent another mechanism for cardiac injury induced by diabetes, secondary to macrophage infiltration, as is evident in other tissues (63). Approaches including targeting the chemokine receptor CCR2 or enhancing heme oxygenase-1 (downstream of Nrf2) favor macrophage polarization towards an M2-like state, mitigating oxidative stress, remodeling, and dysfunction induced by diabetes (64–66).

### 3.2 Cardiac fibrosis in diabetes

A significant population of resident macrophages exists within the heart, which is thought to be related to cardiac electrophysiology and myocardial mitochondrial homeostasis in mouse models (47, 67). When myocardial injury occurs, monocytes are recruited to replace these resident macrophages, performing functions such as phagocytosing dead cells, regulating the inflammatory response, and stimulating the growth of fibroblasts and vascular cells (68). There is ample evidence that macrophages can activate fibroblasts in post-infarction and pressure-overloaded hearts, and in a hyperglycemic environment, induce the expression of adhesion molecules and CC chemokines, which are factors that promote inflammatory responses (69). Therefore, it is reasonable to assume that the recruitment and activation of macrophages may be involved in the process of cardiac fibrosis in diabetes. This hypothesis is supported by several pieces of evidence: infiltration of monocytes and macrophages in the hearts of models of both type 1 and type 2 diabetes (70, 71); cytokines and chemokines produced by macrophages under high glucose conditions can activate fibroblasts, promoting the secretion of fibrotic mediators and proteases (72); exosomes released by activated macrophages are considered to stimulate fibroblasts in diabetic hearts (73); genetic 3 deletion or pharmacological inhibition of the chemokine receptor CCR2 slows cardiac fibrosis in STZ-induced diabetes models (74).

## 4 Epigenetic regulation of macrophages and DCM

The relationship between macrophage epigenetic regulation and diabetic cardiomyopathy (DCM) is complex, engaging a variety of mechanisms. Chronic inflammation and macrophage infiltration play critical roles in the etiology of cardiovascular disorders, including DCM (75). Key epigenetic modifications—DNA methylation, histone adjustments, and RNA alterations—are crucial for macrophage polarization and function. In DCM, inflammation and fibrosis are central to disease progression, with epigenetic mechanisms in macrophages influencing their activation, inflammatory cytokine production, and tissue repair capabilities, thus intensifying myocardial damage and fibrosis (76). Additionally, microRNAs (miRNAs) and long non-coding RNAs (lncRNAs) significantly impact cardiac remodeling in DCM, affecting essential processes like fibrosis and hypertrophy.

### 4.1 lncRNAs

lncRNA encompasses all non-coding RNAs longer than 200 nucleotides, roughly classified into linear lncRNAs and circular RNAs (circRNAs), the latter produced through back-splicing of precursor coding and lncRNA transcripts (77).

#### 4.1.1 DRAIR

DRAIR, or Diabetes Regulated anti-inflammatory RNA, is a long non-coding RNA that plays a significant anti-inflammatory role in inflammation regulation related to diabetes (78), often downregulated in monocytes of diabetic patients (78). Silencing DRAIR inhibits the expression of anti-inflammatory genes in THP1 monocytes and enhances the adhesion of monocytes to ECs and the expression of pro-inflammatory genes such as IL-1β (78). Conversely, overexpression of DRAIR in THP-1 cells increases differentiation of monocytes/macrophages and the expression of anti-inflammatory target genes, while inhibiting pro-inflammatory genes like TNF-α and Fcγ receptor IIIb, suggesting DRAIR regulates the inflammatory phenotype of human monocytes/macrophages through epigenetic mechanisms (78).

#### 4.1.2 Dnm3os

Dnm3os, or dynamin 3 opposite strand, is another lncRNA associated with inflammation responses that promote diabetes and accelerate atherosclerosis. Dnm3os is significantly upregulated in CD14+ monocytes of diabetic patients and observed in various diabetic and atherosclerosis-accelerated mouse models (78, 79). Overexpression of Dnm3os increases the expression of pro-inflammatory genes and phagocytic activity in macrophages, while its silencing produces the opposite effect. Dnm3os interacts with nucleolar proteins, which have protective roles in macrophages. In diabetic conditions, the expression levels of nucleolar proteins decrease, leading to an increase in Dnm3os and promoting the expression of inflammatory genes, indicating Dnm3os plays a role in diabetes and accelerated atherosclerosis (79).

### 4.2 miRNA

miRNAs are non-coding small RNA molecules, about 22 nucleotides in length, playing significant roles in regulating gene expression in eukaryotes by binding to complementary sequences in the 3’UTRs of target mRNAs, causing mRNA degradation or translational repression, thus regulating protein production (80) (81). This regulatory mechanism affects various biological processes including cell differentiation, proliferation, apoptosis, and metabolism, essential for maintaining cellular homeostasis (82). Particularly in the context of diabetic cardiomyopathy, miRNAs such as miR-471 and miR-155 are involved in regulating cell death pathways in macrophages, including apoptosis, necroptosis, and pyroptosis, which are crucial for modulating inflammatory responses and tissue remodeling in the diseased heart.

#### 4.2.1 miR-471

miR-471 is associated with cardiac hypertrophy, identified as a critical negative regulator in the process of mitigating pathological cardiac hypertrophy through preconditioning (83). In bone marrow-derived macrophages (BMDM) of db/db mice and RAW264.7 cells treated with advanced glycation end-products (AGE), miR-471-3p is significantly upregulated, and inhibition of miR-471-3p reduces inflammatory polarization of macrophages. Beyond inflammation, miR-471-3p also influences cell death pathways in macrophages, such as apoptosis and necroptosis, which could have implications for the progression and resolution of cardiac injury in diabetic cardiomyopathy. Bioinformatics analysis identified SIRT1 as a target of miR-471-3p, confirmed by dual-luciferase reporter assay and Western blot, indicating miR-471-3p negatively regulates SIRT1 expression. The SIRT1 agonist resveratrol can downregulate the increased ratio of M1 macrophages induced by AG (83).

#### 4.2.2 miR-155

miR-155 is involved in NLRP3 activation in macrophages by regulating the MEK/ERK/NFκB pathway induced by oxidized low-density lipoprotein (ox-LDL). Its role extends to influencing cell death processes, specifically pyroptosis, in macrophages. Overexpression of miR-155 in apoE-deficient mice via lentivirus accelerates atherosclerosis, indicating that miR-155 may promote inflammatory cell death and contribute to the exacerbation of cardiac damage in diabetic cardiomyopathy (84).

#### 4.2.3 Regulation of macrophage polarization by miRNAs

The role of miRNAs in macrophage polarization is complex and multifaceted. MiR-21, for instance, exhibits dual roles in macrophage polarization; some studies suggest it promotes M2 polarization, while others indicate a preference for M1 polarization (85). This discrepancy underscores the context-dependent nature of miRNA-mediated macrophage polarization. Other miRNAs like miR-9, miR-127, and miR-155 are noted for their roles in promoting M1 polarization, affecting various inflammatory and apoptotic pathways. Conversely, miRNAs such as miR-124, miR-223, and let-7c have been found to favor M2 polarization, highlighting their potential in modulating anti-inflammatory responses (85).

## 5 Macrophage-related therapies for DCM

### 5.1 Targeted therapies

miRNAs regulate macrophage phenotypes in various disease processes. For example, miR155 directly blocks IL-13-induced M2 macrophage phenotype by inhibiting IL-13Rα1 expression (86). Imbalance of M1/M2 exacerbates cardiomyopathy in orchiectomized diabetic mice. Intravenous injection of miR155-AuNP, where thiol-modified antago-miR155 covalently binds to gold nanoparticles (AuNP), preferentially delivers nucleic acids to macrophages through phagocytosis. Delivery of antago-miR155 *in vivo* reduces apoptosis and restores cardiac function by increasing the M2 ratio and reducing inflammation (87).

In diabetic rat hearts, levels of substance P (SP), a sensory neuropeptide, are decreased, resulting in the loss of cardioprotective effects post-ischemic treatment. SP promotes the transition to a reparative M2 macrophage phenotype, which in mice is characterized by markers such as arginase 1 and IL-10. It is important to note that arginase 1 serves as a marker for the M2a phenotype predominantly in murine models, and its expression and functional role may differ in human macrophages (29, 30). Leprdb/db mice show increased LV M1 phenotype macrophages and an elevated M1/M2 ratio. Substitution of SP in Leprdb/db mice restores a favorable M1 to M2 balance, suggesting SP deficiency makes diabetic hearts prone to fibrosis. The anti-fibrotic effects of SP substitution involve direct effects on cardiac fibroblasts and macrophages to counter adverse phenotypic changes (88).

Bone morphogenetic protein (BMP)-7, a novel mediator of monocyte polarization, activates infiltrating monocytes into anti-inflammatory M2 macrophages, inhibiting apoptosis and fibrosis and improving cardiac function. Additionally, considering the nonresolving nature of inflammation in Type I diabetes, efferocytic macrophages could play a crucial role in mitigating the persistent inflammation associated with DCM. Efferocytic macrophages, adept at clearing apoptotic cells, might help to promote the resolution of inflammation, thus offering a potential therapeutic strategy for DCM. Employing pro-resolving factors, as highlighted by Nathan C et al. and Saas P et al., could further facilitate this process by enhancing the efferocytosis and restoring homeostasis (89–92).

### 5.2 Natural food components

#### 5.2.1 Triptolide

Triptolide, derived from the root of the Tripterygium wilfordii plant, harbors triptolide as its most pivotal bioactive constituent. This herb, entrenched in traditional Chinese medicine, has been employed for over three decades in the management of glomerulonephritis and immune-mediated disorders, such as complex nephritis and systemic lupus erythematosus, attributed to its immunosuppressive, antiproliferative, and anti-inflammatory properties (93). Triptolide effectively protects against DCM, preventing immune dysregulation, myocardial inflammation, and cardiac fibrosis in diabetic rats, thereby alleviating left ventricular dysfunction. Triptolide’s protective effects on cardiac tissue may be attributed to inhibiting the TLR4-induced NF-κB/IL-1β immune pathway, the NF-κB/TNF-α/VCAM-1-mediated inflammatory pathway, and downregulating TGF-β1/α-SMA/Vimentin involved in cardiac fibrosis, indicating potential beneficial effects of triptolide on DCM (93).

#### 5.2.2 Ginsenoside RG1

Ginsenoside Rg1, one of the paramount components found in Panax ginseng (94), is acclaimed for its cardiovascular protective effects, primarily through the inhibition of cellular apoptosis and mitigation of oxidative stress (95). Research focusing on the cardioprotective mechanisms of ginsenoside Rg1 predominantly targets conditions such as hypertrophic cardiomyopathy, acute myocardial infarction, and myocardial ischemia/reperfusion injury (96–98). Ginsenoside RG1 improves DCM in diabetic rats by inhibiting endoplasmic reticulum stress-induced apoptosis and alleviating oxidative stress and myocardial cell apoptosis induced by streptozotocin in diabetic rats. Ginsenoside RG1 has been shown to be an effective regulator of MSCs. RG1-induced MSC-secreted exosomal circNOTCH1 alleviates DCM by activating NOTCH signaling, promoting macrophage M2 polarization pathways (99).

#### 5.2.3 Curcumin

Curcumin, the principal bioactive constituent of turmeric, derived from the rhizomes of the Curcuma longa plant (100), exhibits multifaceted pharmacological activities, including antioxidative, free radical scavenging, anti-inflammatory, antineoplastic, and antimicrobial properties (101). Previous investigations have elucidated that curcumin treatment ameliorates diabetic cardiomyopathy (DCM) through attenuation of reactive oxygen species (ROS), apoptosis, and inflammation, thereby significantly enhancing cardiac function and mitigating fibrosis (101). JM-2, a novel curcumin analog, has been demonstrated to effectively prevent cardiac functional and structural aberrations, reducing cardiac inflammation and fibrosis. The therapeutic administration of JM-2 mitigates DCM by inhibiting the activation of NF-κB in cardiac tissue. Moreover, JM-2 treatment completely abrogated the elevation of pro-inflammatory cytokines and macrophage infiltration in models of type 1 and type 2 diabetes mellitus. RNA sequencing analyses have revealed that the anti-inflammatory activity of JM-2 correlates with the suppression of NF-κB activation. *In vitro*, JM-2 attenuates high glucose-induced cardiomyocyte hypertrophy and fibrosis, concurrently inhibiting the activation of NF-κB (58).

#### 5.2.4 Puerarin

Puerarin, also known as dihydroxyisoflavone, isolated from the roots of the traditional Chinese medicinal herb Pueraria lobata, possesses a spectrum of beneficial activities against cardiovascular disorders, including myocardial infarction (102). Puerarin has been reported to prevent diabetic cardiomyopathy both *In vitro* and *in vivo* through the inhibition of inflammatory pathways (103). Studies have shown that puerarin mitigates cardiomyocyte and macrophage necroptosis via the inhibition of the NLRP3-Caspase-1-GSDMD pathway mediated by the P2X7 receptor (104). *In vitro*, puerarin treatment significantly reduces D-glucose-induced cell death in H9C2 cardiomyocytes and exerts anti-inflammatory effects on RAW264.7 macrophages. The P2X7 receptor-mediated necroptosis is posited as a pivotal mechanism in the therapeutic action of puerarin against diabetic cardiomyopathy (104).

#### 5.2.5 Syringaresinol

Syringaresinol (SYR), a phenolic compound prevalent in various cereals and medicinal plants, is noted for its anti-inflammatory and antioxidative pharmacological properties (105). Oral administration of SYR bi-daily for eight weeks in STZ-induced type 1 diabetic mice significantly ameliorated cardiac dysfunction and prevented cardiac hypertrophy and fibrosis. SYR also inhibited macrophage infiltration and oxidative stress biomarkers without impacting hyperglycemia and body weight. In neonatal cardiomyocytes, SYR effectively reduced high glucose-induced apoptosis and fibrosis, and incubation with SYR alleviated inflammatory response and oxidative stress. Mechanistically, SYR may exert protective effects by restoring suppression of antioxidant kelch-like ECH-associated protein 1 (Keap1)/nuclear factor-E2-related factor 2 (Nrf2) system and abnormal activation of transforming growth factor-β (TGF-β)/mothers against decapentaplegic homolog (Smad) signaling pathway *In vitro* and in vivo (106).

#### 5.2.6 Aza resveratrol–chalcone compounds

Aza-resveratrol-chalcone compounds, considered precursors to resveratrol and chalcone, are ubiquitously present in edible plants and have been reported to exhibit diverse pharmacological effects on vascular diseases, cancer, viral infections, and inflammation *In vitro* and *in vivo* (107, 108). A synthesized derivative, 6b, effectively inhibited LPS-induced macrophage inflammatory response. Inflammation and oxidative stress are implicated in the pathogenesis of DCM, and studies have demonstrated that 6b prevents inflammation, oxidative stress, hypertrophy, fibrosis, and apoptosis in DCM through blocking NF-κB nuclear translocation and activating the Nrf2 pathway both *In vitro* and *in vivo* (109).

#### 5.2.7 Isoliquiritigenin

**Figure 3:**
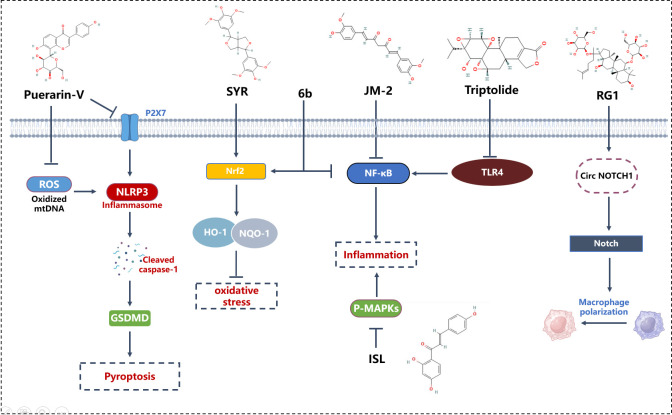
Mechanism of phytomedicine in improving DCM through macrophage modulation. 6b, Aza resveratrol–chalcone compounds; GSDMD, Gasdermin D; HO-1, Heme Oxygenase-1; ISL, Isoliquiritigenin; JM-2, Curcumin analog; NF-κB, Nuclear Factor kappa-light-chain-enhancer of activated B cells; NLRP3, NOD-, LRR- and pyrin domain-containing protein 3; NQO-1, NAD(P)H Quinone Dehydrogenase 1; Nrf2, Nuclear Factor Erythroid 2–Related Factor 2; P-MAPKs, Phosphorylated Mitogen-Activated Protein Kinases; RG1, Ginsenoside RG1; ROS, Reactive Oxygen Species; SYR, Syringaresinol; TLR4, Toll-Like Receptor 4.

Isoliquiritigenin (4,2’,4’-trihydroxychalcone) (ISL), a widespread chalcone present in Glycyrrhiza uralensis Fisch., Dianthus chinensis L., and Astragalus membranaceus Moench, is renowned for its anti-inflammatory, antioxidant, and antitumor properties (110, 111). It was generally recognized than ISL possess pharmacological properties, such as anti-inflammatory, antioxidant and antitumor activities (112). Research has indicated that ISL mitigates diabetic cardiomyopathy by suppressing high glucose-induced inflammatory responses and oxidative stress (113). ISL also possesses anti-inflammatory effects on LPS-stimulated macrophages and inhibits macrophage infiltration in cardiac tissue (113, **Figure 3**).

## 6 The modulatory effects of existing antidiabetic drugs on macrophages

### 6.1 SGLT2 inhibition

Sodium-glucose cotransporter-2 (SGLT2) is primarily expressed in the S1 and S2 segments of the renal proximal tubules, the region responsible for reabsorbing 90% of the filtered glucose (114). Recently, SGLT-2 inhibitors have been developed as novel therapeutic drugs for T2DM patients. Clinical trials suggest that SGLT2 inhibitors may offer benefits beyond diabetes treatment, including reduced risk of hospitalization for heart failure or cardiovascular death. These benefits are now available to individuals with and without T2DM (115). In a randomized controlled trial, dapagliflozin reduced heart failure by 27% in T2DM (type 2 diabetes mellitus) patients with cardiovascular disease. The effectiveness was 35% for empagliflozin and 33% for canagliflozin (116). SGLT2 inhibitors might benefit the cardiovascular system by reducing adipose tissue-mediated inflammation and pro-inflammatory cytokine production, promoting ketone bodies as metabolic substrates for the heart and kidneys, decreasing oxidative stress, lowering serum uric acid levels, and reducing glomerular hyperfiltration and albuminuria (117).

A prospective, randomized, open-label, active-controlled, two-arm parallel intervention study in T2DM patients with high cardiovascular risk treated with SGLT2 inhibitors or sulfonylureas for 30 days analyzed the activation of NLRP3 inflammasomes in macrophages. Although the glucose-lowering ability of SGLT2 inhibitors was similar to sulfonylureas, they showed greater reduction in IL-1β secretion, accompanied by increased serum β-hydroxybutyrate (BHB) and decreased serum insulin. Ex-vivo experiments with macrophages confirmed the inhibitory effect of high BHB and low insulin levels on NLRP3 inflammasome activation (118).

Studies show that Empagliflozin reduces the accumulation of M1 polarized macrophages while inducing an anti-inflammatory M2 phenotype in adipose and liver macrophages, alleviating obesity-induced inflammation and insulin resistance. Empagliflozin increases energy expenditure, thermogenesis, and expression of uncoupling protein 1 in brown adipose tissue, inguinal and epididymal white adipose tissue (WAT). Furthermore, Empagliflozin reduces the accumulation of M1 polarized macrophages while inducing an anti-inflammatory M2 phenotype in WAT and liver, lowering plasma TNFα levels and alleviating obesity-related chronic inflammation (119).

In a 5/6 nephrectomy rat model, the beneficial effects of Empagliflozin on renal function and morphology are partly attributed to inhibition of CD206^+^ CD68^+^ M2 macrophage polarization by targeting the mTOR and mitochondrial autophagy pathways and attenuating inflammation signals from CD8^+^ effector T cells (120).

Research indicates that Canagliflozin can also inhibit atherosclerosis (AS). In macrophages, Canagliflozin enhances autophagy, promoting cholesterol efflux and reducing intracellular lipid droplet concentration. More importantly, Canagliflozin increases macrophage autophagy in atherosclerotic plaques of ApoE^-/-^ mice, suggesting it could help inhibit the development of AS. Overall, these actions of Canagliflozin reduce the formation of atherosclerotic lesions, providing new insights into the potential mechanisms by which SGLT2 inhibitors slow the progression of this disease (121).

### 6.2 GLP1 receptor agonists

Liraglutide (LRG), a glucagon-like peptide 1 analog (GLP1A), can reduce weight in T2DM. In a randomized, placebo-controlled trial, patients with type 2 diabetes mellitus, devoid of prior cardiovascular disease, demonstrated a reduction in early diastolic left ventricular filling and left ventricular filling pressure upon treatment with liraglutide, as opposed to the addition of a placebo to the standard therapy. This effect ameliorated left ventricular load. Concurrently, left ventricular systolic function diminished yet remained within normal limits (122).

Lixisenatide has been reported to prevent acute cardiovascular events in Type 2 Diabetes (123). Treatment with Lixisenatide in Apoe^-/-^ Irs2^+/-^ mice (a mouse model of insulin resistance, metabolic syndrome, and AS resulted in more stable atherosclerotic plaques with less inflammatory infiltration, reduced necrotic core, and a thicker fibrous cap. Lixisenatide reduced the secretion of the pro-inflammatory cytokine IL-6 while enhancing the activation of Signal Transducer and Activator of Transcription (STAT)3, a determinant for M2 macrophage differentiation. The activation of STAT1, necessary for M1 phenotype, was also diminished. The study suggests that Lixisenatide reduces the size and instability of atherosclerotic plaques in Apoe^-/-^ Irs2^+/-^ mice by reprogramming macrophages to the M2 phenotype, thereby reducing inflammation (124).

Exendin-4 has restorative effects on tubular damage in a STZ-induced diabetic model. Exendin-4 improved proteinuria, glomerular hyperfiltration, glomerular hypertrophy, and mesangial matrix expansion in diabetic rats without altering blood pressure or body weight. Exendin-4 also prevented macrophage infiltration, reduced the protein levels of ICAM-1, and type IV collagen, and decreased oxidative stress and nuclear factor-κB activation in renal tissue (125). Exendin-4 acts directly on GLP-1 receptors and attenuates the release of pro-inflammatory cytokines in macrophages and the production of ICAM-1 on glomerular ECs (126).

Additionally, Exendin-4 has acute cardioprotective effects and benefits experimental/clinical heart failure. Exendin-4 prevents post-MI remodeling through preferential action on inflammation and the extracellular matrix (127). Exendin-4 and insulin improved metabolic parameters in diabetic mice after 12 weeks, but only Exendin-4 reduced diastolic dysfunction and interstitial fibrosis while altering ECM gene expression. Exendin-4 prevents ECM remodeling and diastolic dysfunction in experimental diabetes by preferentially preventing paracrine communication between infiltrating macrophages and resident fibroblasts, suggesting that cell-specific targeting of GLP-1 signaling might be a viable treatment strategy in this context (128).

### 6.3 DPP4 inhibitors

Dipeptidyl peptidase-4 (DPP-4) inhibitors, employed primarily to manage type 2 diabetes, function by inhibiting the degradation of glucagon-like peptide-1 (GLP-1), thus facilitating glycemic control. Beyond their role in glucose regulation, DPP-4 inhibitors have been substantiated to exert cardioprotective effects in ischemic heart models (129, 130). Alogliptin inhibits monocyte activation/chemotaxis, playing an anti-atherosclerotic role and reducing inflammation. In a male LDLR^-/-^ mouse model fed a high-fat diet, Alogliptin reduced visceral adipose tissue macrophage content (adipose tissue macrophages; CD11b^+^, CD11c^+^, Ly6C^hi^) while upregulating CD163. DPP-4 is highly expressed in bone marrow-derived CD11b^+^ cells, and Alogliptin downregulates pro-inflammatory genes in these cells. DPP-4i reduced aortic plaques and significantly decreased plaque macrophages. *In vitro*, Alogliptin blocked monocyte migration and actin polymerization through a Rac-dependent mechanism and prevented the migration of labeled monocytes responsive to exogenous TNF-α and DPP-4 to the aorta (131).

### 6.4 Thiazolidinediones

Thiazolidinediones (TZDs) represent a class of drugs that directly target insulin resistance through activation of PPARγ agonists. These agents are efficacious antihyperglycemic medications with numerous potential cardiovascular advantages (132, 133). The prospective pioglitazone clinical trial in major vascular events, known as the PROactive study, indicated that TZDs, specifically pioglitazone, reduced the risk of the primary endpoint—a composite of disease and surgery-related outcomes—by 10% (134). TZDs are ligands for Peroxisome Proliferator-Activated Receptor-γ (PPAR-γ) and possess anti-inflammatory effects independent of their insulin-sensitizing actions. Pioglitazone (PIO) treatment did not change blood glucose and HbA1c. PIO reduced urinary albumin excretion and glomerular hypertrophy, inhibiting expression of transforming growth factor (TGF)-β, type IV collagen, ICAM-1, and macrophage infiltration in diabetic rat kidneys. PPAR-γ is expressed in glomerular ECs of diabetic kidneys and cultured glomerular ECs. HG conditions increased ICAM-1 expression and NF-κB activation in cultured glomerular ECs. PIO, ciglitazone, and pyrrolidine dithiocarbamate (a NF-κB inhibitor) reduced these changes. PIO’s preventive effects are likely mediated through its anti-inflammatory actions, including inhibition of NF-κB activation, ICAM-1 expression, and macrophage infiltration in diabetic kidneys (135). Additionally, PIO reduces inflammation in high-fat diet diabetic mice by inhibiting AGE-induced classical macrophage activation (136).

Rositiglitazone (ROSI), as a modulator of macrophage polarization, can mitigate renal calcium oxalate (CaOx) deposition by alleviating oxidative stress and inflammatory responses. CaOx crystals upregulate NADPH oxidase p47phox, leading to excessive production of ROS in RTECs. The pro-inflammatory action of macrophages further intensifies this effect (137). ROSI significantly reduces renal crystal deposition, tubular damage, crystal adherence, cellular apoptosis, oxidative stress, and inflammatory responses. *In vitro*, ROSI markedly inhibits renal injury, cellular apoptosis, and crystal adhesion in HK-2 cells, and significantly transforms COM-stimulated M1 macrophages into M2 macrophages, exhibiting anti-inflammatory effects. Moreover, ROSI significantly suppresses oxidative stress by promoting the Nrf2/HO-1 pathway in HK-2 cells. These findings suggest that ROSI can ameliorate tubular damage caused by oxidative stress and inflammatory responses by inhibiting M1 macrophage polarization and promoting M2 macrophage polarization (138).

### 6.5 Biguanide

Metformin is among the most frequently employed antidiabetic agents in clinical practice (139). Research has substantiated its efficacy in ameliorating cardiac fibrosis and oxidative stress, as well as in augmenting the levels of mitochondrial antioxidant enzymes (140). Furthermore, metformin has been reported to enhance glycemic control and mitigate the effects of diabetic (140). The therapeutic potential of Metformin in treating obese/diabetic patients is linked to its capacity to counteract insulin resistance. LPS activate M1 macrophages, promoting paracrine activation of HIF-1α in brown adipocytes, subsequently diminishing insulin signaling and glucose uptake, as well as β-adrenergic sensitivity. The addition of Metformin to M1 polarized macrophages attenuates these indications of brown adipocyte functional impairment. At the molecular level, Metformin induces the degradation of HIF1α by inhibiting mitochondrial complex I activity, thereby curtailing the HIF1α-mediated inflammatory program in macrophages and reducing oxygen consumption in a ROS-independent manner. In summary, Metformin’s inhibition of HIF1α-dependent pro-inflammatory programs in macrophages may indirectly elicit beneficial effects on insulin-mediated glucose uptake and β-adrenergic responses in brown adipocytes (141).

## 7 Conclusion

This article contributes to the evolving understanding of the complex role of macrophages in diabetic cardiomyopathy (DCM), indicating their potential as therapeutic targets. It has highlighted intricate mechanisms through which macrophages influence DCM, particularly focusing on epigenetic modulation and their interactions within the cardiac microenvironment. Therapeutically, modulating macrophage activity appears to hold promise in addressing DCM. Strategies such as epigenetic adjustments, the targeted manipulation of macrophage functions, and the exploration of natural substances with anti-inflammatory effects are being considered. These methods aim to subtly shift macrophage behavior towards a more reparative phenotype, potentially easing myocardial impairment in DCM.

Moreover, the review suggests that a deeper insight into macrophage dynamics in DCM could lead to more balanced therapeutic strategies. These would ideally leverage the protective roles of macrophages while minimizing their harmful aspects. Incorporating macrophage-focused treatments into the broader management strategy for DCM could offer a valuable pathway for research and might improve clinical outcomes. In closing, while targeting macrophages in DCM shows potential, it is presented here as a promising but exploratory approach. The common mechanisms identified, including the regulation of inflammation, epigenetic changes, and improved cell signaling, warrant further investigation. Continuing research in this direction may provide a foundation for developing nuanced and effective macrophage-targeted therapies, aiming to alleviate the impact of diabetic cardiomyopathy.

## Author contributions

SZ: Writing – original draft, Writing – review & editing. XZ: Writing – review & editing. YC: Conceptualization, Writing – review & editing. ZW: Formal analysis, Resources, Writing – review & editing. PS: Conceptualization, Writing – review & editing. QN: Writing – original draft.

## Glossary

**Table:**

AGEs	advanced glycation end products
AGM	aorta-gonad-mesonephros
ALA	Alantolactone
AS	atherosclerosis
AT-II	angiotensin-II
BHB	β-hydroxybutyrate
BMP	Bone Morphogenetic Protein
BMDMs	bone marrow-derived macrophages
BRB	blood-retinal barrier
C3G	Cyanidin-3-O-glucoside
CaOx	calcium oxalate
CCL	C-C Motif Chemokine Ligands
CCR2	C-C chemokine receptor 2
CSF	colony-stimulating factors
CVD	cardiovascular disease
DCM	diabetic cardiomyopathy
DKD	Diabetic Kidney Disease
DM	Diabetes Mellitus
DN	diabetic nephropathy
DPN	diabetic peripheral neuropathy
DR	diabetic retinopathy
ESRD	end-stage renal disease
FA	Ferulic Acid
FFA	free fatty acid
FGFR3	Fibroblast Growth Factor Receptor 3
FTO	Fat Mass and Obesity-Associated
GCL	ganglion cell layer
GLP1A	glucagon-like peptide 1 analog
GSIS	glucose-stimulated insulin secretion
GSPE	Grape Seed Proanthocyanidin Extract
HIF-1α	Hypoxia-Inducible Factor 1 alpha
HFHSD	high-fat high-sugar diet
HG	high glucose
HSCs	hematopoietic stem cells
IAPP	Islet amyloid polypeptide
ICAM-1	Intercellular Adhesion Molecule-1
IFN-γ	interferon-γ
IL	interleukins
IPL	inner plexiform layer
JNK	Jun N-terminal kinase
LPS	lipopolysaccharides
LRG	Liraglutide
MAPK	mitogen-activated protein kinase
MCP	monocyte chemoattractant protein
MDA	Malondialdehyd
MNCV	Motor Nerve Conduction Velocity
MMP	matrix metalloproteinases
PEDF	pigment epithelium-derived factor
PERO	Pioglitazone
PPAR-γ	Peroxisome Proliferator-Activated Receptor-γ
RPE	retinal pigment epithelium
RBM15	RNA-binding motif protein 15
ROS	reactive oxygen species
ROSI	Rositiglitazone
RTECs	Renal Tubular Epithelial Cells
SGLT2	Sodium-glucose cotransporter-2
SC	Scutellarin
SIRT6	Sirtuin-6
SOD	Superoxide Dismutase
SP	Substance P
STAT	Signal Transducer and Activator of Transcription
STZ	streptozotocin
T2DM	Type 2 Diabetes Mellitus
TEC	Tectorigenin
TGF-β	transforming growth factor-β
Tim-3	T-cell immunoglobulin mucin domain 3
TLR4	Toll-like receptor 4
TNF-α	tumor necrosis factor-α
TP	Triptolide
TZDs	Thiazolidinediones
VCAM-1	Vascular Cell Adhesion Molecule-1
VEGFA	vascular endothelial growth factor-A
WAT	white adipose tissue.
